# The Serine/Threonine-Protein Phosphatase 1 From *Haemonchus contortus* Is Actively Involved in Suppressive Regulatory Roles on Immune Functions of Goat Peripheral Blood Mononuclear Cells

**DOI:** 10.3389/fimmu.2018.01627

**Published:** 2018-07-16

**Authors:** Muhammad Ehsan, WenJuan Wang, Javaid Ali Gadahi, Muhammad Waqqas Hasan, MingMin Lu, YuJian Wang, XinChao Liu, Muhammad Haseeb, RuoFeng Yan, LiXin Xu, XiaoKai Song, XiangRui Li

**Affiliations:** Department of Preventive Veterinary Medicine, College of Veterinary Medicine, Nanjing Agricultural University, Nanjing, China

**Keywords:** *Haemonchus contortus*, serine/threonine phosphatase, peripheral blood mononuclear cells, cytokines, immune responses

## Abstract

Serine/threonine-protein phosphatases (STPs), as integral constituents of parasitic excretory/secretory proteins, are assumed to be released during the host–parasite interactions. However, knowledge about these phosphatases and their immunoregulatory and immune protective efficiencies with host peripheral blood mononuclear cells (PBMCs) is scant. In this study, an open reading frame of STP from *Haemonchus contortus* designated as HcSTP-1 was amplified and cloned using reverse-transcription-polymerase chain reaction (RT-PCR) method. The 951-bp nucleotides sequence was encoded to a protein of 316 amino acid residues, conserved in characteristics motifs GDXHG, GDYVDRG, GNHE, HGG, RG, and H. The HcSTP-1 protein was detected at approximately 35 kDa as recombinant protein fused in an expression vector system and resolved on sodium dodecyl sulfate-polyacrylamide gel electrophoresis. Immunohistochemically, HcSTP-1 was found to be localized in both male and female adult worm sections. Using immunofluorescence assay, the binding activity of rHcSTP-1 was confirmed on surface of goat PBMCs, which resulted in expression of multiple cytokines and various immunoregulatory activities *in vitro*. The RT-PCR results showed that mRNA level of interleukin-2, TGF-β1, IFN-γ, and IL-17 (with 10 µg/ml) was upregulated and IL-10 was decreased. However, IL-6 showed no change after PBMCs incubated with rHcSTP-1 protein. Further functional analysis showed that migratory activity of cells, intracellular nitrite production (NO), and apoptotic efficiency of PBMCs were elevated at significant level, whereas the proliferation of goat PBMCs and monocytes-associated major histocompatibility complex (MHC)-I and MHC-II expressions were decreased significantly at concentration-dependent fashion. Our results showed that the HcSTP-1 protein engaged in vital suppressive regulatory roles on host immune cells, which might represent a potential molecular target for controlling *H. contortus* infection in future.

## Introduction

Among gastrointestinal nematodes, *Haemonchus contortus* (barber pole worm) is most important parasite, responsible for health and economic problems in livestock industry worldwide (1). In severely infected animals like sheep and goats, the adult parasite penetrates in abomasal mucosa to feed on blood, results in anemia, loss of body weight and growth, and even death of the animals (2). Existing approaches for control of nematode parasites including *H. contortus*, mainly through widespread antihelmintics have resulted in stern drug resistance in domestic livestock (3, 4). Hence, the emergence of drug resistance in *H. contortus* demands for discoveries of novel anti-parasite drugs, vaccines, and development of immunological control strategies against nematode infections. In this regard, a comprehensive insight into the developmental biology of *H. contortus* at the molecular level might pinpoint some key antigens as new drug targets for parasite control (5).

The serine/threonine-protein phosphatase 1 (STP-1) belong to phosphoprotein phosphatase (PPP) family, and protein phosphatases (PPs) is largest class which is divided functionally into two major groups: first serine/threonine phosphatases (commonly cytoplasmic structural component, concerned with transcriptional activity and signal transduction) and second tyrosine phosphatases (typically bound with membrane and implicated in receptor-arbitrated signal transduction) (6). PPs can also be divided into four subclasses, such as PP1, PP2A, PP2B, and PP2C, based on substrate and inhibitory specificities (7). Furthermore, it was reported that PPs have been implicated in range of biological functions, such as exocytosis and apoptosis, neuronal activity, ion channel electrophysiology, and cell cycle (8–12).

Previously, various STPs from number of parasites have been identified and characterized, including those of *Oesophagostomum dentatum, Trichostrongylus vitrinus, Toxoplasma gondii, Toxocara canis*, and *Plasmodium falciparum* (13–17), and also suggested that STP-1 from *H. contortus* play a key role in some biological processes, such as spermatogenesis (18). However, information regarding the functions of STPs in parasitic nematode *H. contortus* is scant.

In our previous comparative proteomics analysis of excretory/secretory proteins (ESPs) from *H. contortus* (HcESPs), 59 proteins were identified at early and late adult developmental stages of parasite and amongst all, serine/threonine-protein phosphatase (STPs) were recognized as vital interacting proteins bind to the goat peripheral blood mononuclear cells (PBMCs) *in vivo* (19). The main objectives of current study were to clone and characterize a stage-specific gene from *H. contortus* designated as HcSTP-1, to express its recombinant protein in an expression vector system, and to evaluate potential immune regulatory roles of this protein (rHcSTP-1) with goat PBMCs *in vitro*. Our findings will provide valuable bases to better understand the biology of this parasite and to develop effective drugs and useful vaccines, which could be helpful in the prevention and control of *H. contortus* infection.

## Materials and Methods

### Ethics Statement

All animals and laboratory experiments were strictly performed in accordance with the recommendations of the Animal Ethics Committee, Nanjing Agricultural University, China. All experimental procedures used in this study were permitted by the science and Technology Agency of Jiangsu Province [ID: SYXK (SU) 2010-0005].

### Animals, Parasites, and PBMC Isolation

The native crossbred goats (3–6 months old) were housed indoor, provided with hay and corn (whole shelled) and water *ad libitum*. The anti-parasitic drug, levamisole (8 mg/kg body weight) was used with 2 weeks interval to keep goats free from naturally acquired helminth infection. The fecal samples were checked twice per week, using standard parasitological techniques and goats with no sign of helminths infection were used in subsequent experiments.

Sprague-Dawley (SD) rats with average body weight of 150 g, were bought at Experimental Animal Center of Jiangsu, PR China (Certified: SCXK 2008-0004). The rats were raised in microbe free condition.

The *H. contortus* strain was maintained by serial passage in helminth-free goats, at laboratory of immunology and molecular parasitology, Nanjing Agricultural University. The parasite eggs, larvae, and adults worms were collected and preserved according to the methods stated previously (20). The blood samples were taken from jugular vein of dewormed goats and PBMCs were collected by gradient centrifugation method (21). The PBMCs were cultured as the procedure stated previously (22).

### Cloning and Sequence Analysis *H. contortus* STP-1 Gene

The freshly adults *H. contortus* parasites were collected from abomasum of the infected goats and the extraction of RNA was carried out using Trizol method according to the previously described procedure (23), followed by cDNA synthesis as per the manufacturer’s guidelines of cDNA synthesis Kit (TaKaRa Biotech), and was preserved at −20°C for down-stream applications.

The open reading frame of HcSTP-1 was amplified by reverse-transcription-polymerase chain reaction (RT-PCR), from conserved domain sequences of *H. contortus* STP-1 gene (GenBank: GQ280010.1). For the subsequent cloning, underlined *BamH* I and *Xho* I restriction enzyme were inserted at the 5′-end of forward primer: (5′-GGATCCATGGACCCTACTCAAT-3′), and reverse primer: (5′-CTCGAGTTATTGACAAGGTGGAGC-3′). The amplified PCR fragment was electrophoresed and eluted with Gel purification Kit (Omega, USA) in accordance with kit protocol. The eluted product was then inserted into pMD19-T cloning vector (TaKaRa Biotechnology, China). The recombinant plasmid pMD19-T/HcSTP-1 was inserted into DH5α competent strain of *Escherichia coli* and cultured in Luria–Bertini medium with ampicillin (100 µg/ml). The positive clones followed by sequencing (Invitrogen Biotech, Shanghai, China) were validated by sequence analysis online using Blast program.^1^ The obtained nucleotide sequence was translated into amino acids sequence and aligned and compared with different nematode species using GENETYX Version 7.0.9 software. In addition, a number of online approaches/programs were used to detect N-terminal signal peptides/http://www.cbs.dtu.dk/services/SignalP/, Transmembrane protein prediction/http://www.cbs.dtu.dk/services/TMHMM/, T cell motifs/DNAstar: EditSeq, Protean, B cell epitopes/http://tools.immuneepitope.org/tools/bcell/iedb_input, and GPI modification Site Prediction/http://mendel.imp.ac.at/sat/gpi/gpi_server.html.

### Expression and Purification of Recombinant HcSTP-1 Protein

The recognized recombinant pMD19-T/HcSTP-1 plasmid was digested with dual restriction enzymes *BamH* I and *Xho* I and ligated into prokaryotic expression vector pET32a (+) (Novagen, USA). Finally, the successful cloned STP-1 gene in a recombinant expression vector was sequenced to confirm its placement in the accurate reading frame. The recombinant plasmid pET32a (+)-HcSTP-1 was transferred into *E. coli* strain (BL21) and induced with 1 mM isopropyl-β-d-thiogalactopyranoside (IPTG; Sigma-Aldrich) after the OD_600_ of the culture reached 0.6 at 37°C. The cell pellet after centrifugation was lysed using 10 µg/ml of lysozyme (Sigma-Aldrich) followed by sonication, and was resolved on 12% (w/v) sodium dodecyl sulfate-polyacrylamide gel electrophoresis (SDS-PAGE). The purification of recombinant protein was carried out according to the manufacturer’s instructions of Ni^2+^-nitrilotriacetic acid (Ni-NTA) column (GE Healthcare, USA). The histidine-tagged protein (empty pET32a) used as control protein in multiple assays in this study was purified and expressed similar to the procedure described for rHcSTP-1 protein and determined at 12% SDS-PAGE after Coomassie blue staining and quantified by Bradford method (24).

### Immuno-Blot Analysis

Polyclonal antibodies against recombinant protein were generated from SD rats by subcutaneous injection of 300 µg of rHcSTP-1 protein mixed equally with Freund’s complete. After 2 weeks of first immunization, rats were injected three times at 1 week interval with same protein and Freund’s incomplete mixture. The sera were collected after 10 days of last injection and preserved at −80°C for later use. The sera against *H. contortus* parasites were collected from naturally infected goats (25), and sera collected from normal goats or rats were used as negative control.

The SDS-PAGE products of recombinant HcSTP-1 and soluble/ES products from adult parasites, were shifted nitrocellulose membrane (Millipore, USA) for western blot analysis. After blocked with 5% (w/v) skimmed milk powder in TBST (TBS with 0.5% Tween-20) at 37°C for 2 h, the strips were incubated with anti-*H*. *contortus* sera from goats or rat anti-sera against rHcSTP-1 (1:300 dilutions) as first antibody for treatment groups and normal goat serum or normal rat serum for control groups at 4°C overnight. Then, the membranes were incubated with the secondary antibody, HRP-conjugated goat anti-rat IgG (Santa Cruz, USA) in TBST (1:3,000 dilutions) at 37°C for 2 h. At end, the immunoreactions were visualized within 3–5 min, as per defined protocol of DAB Horseradish Peroxidase Color Development Kit (Beyotime Biotechnology).

### Localization Assay

The mature parasites were suspended in TISSUE-TEK^®^ O.C.T. compound (SAKURA, Torrance, CA, USA) and snap-frozen in liquid nitrogen. By using cryotome (CM1950, Wetzlar, Germany), worms were cut into sections with 10-µm thickness and fixed on poly-l-lysine hydrobromide glass slides. Non-specific bindings were confiscated by treating the slides with 10% normal goat serum, followed by incubation with rat-anti-STP-1 antiserum (1:300 dilutions) or normal rat serum as first antibody and Cy3-labeled Goat Anti-Rat IgG (1:3,000 dilutions) as second antibody (Beyotime, Shanghai, China) at 37°C for 2 h in each step. For DNA staining, the sections were marked with 1.5 µM 2-(4-amidinophenyl)-6-indolecarbamidine dihydrochloride (DAPI: Sigma, MO, USA) for 5 min. Finally, Anti-Fade Fluoromount Medium (Beyotime, Shanghai, China) was used and sections were examined under confocal microscope.

### Binding of rHcSTP-1 on the Surface of Goat PBMCs

Freshly collected PBMCs, after washed with phosphate buffer saline (PBS: Ca^2+^/Mg^2+^-free, pH 7.4), were maintained at density of 1 × 10^5^ cells/ml in cell culture medium (RPMI 1640), containing 10% fetal bovine serum (FBS), 100 U/ml penicillin and 100 mg/ml streptomycin (Gibco, Life Technology), and incubated with rHcSTP-1 or control protein pET32a or control buffer (PBS) at constant temperature (37°C) and humidity (5% CO_2_) for 4 h. Protein binding at surface of goat PBMCs were confirmed by immunofluorescence assay (IFA). Briefly, PBMCs after being washed and stabled with 4% paraformaldehyde on poly-l-lysine-treated slides were then permeabilized using 1% TritonX-100 in PBS at normal temperature. The PBMCs were subjected to primary antibodies rat anti-rHcSTP-1-IgG (1:300 dilutions) or normal rat serum followed by Cy3-coupled goat anti-rat IgG (Beyotime, China) (1:3,000 dilutions) as second antibody for 1 h at 37°C temperature. At last, the slides were stained in darkness with DAPI (Sigma, USA) for 5 min, and subjected to Anti-Fade Fluoromount solution (Beyotime, China) before laser scanning confocal microscopic examination (LSM710, Zeiss, Jena, Germany) at 100× magnifications.

### Detection of Cytokine Transcripts by RT-PCR

The PBMCs were stimulated with Concanavalin A (ConA/10 μg/ml), and treated with rHcSTP-1 (10, 20, and 40 µg/ml) or pET32a protein (10 µg/ml) and control buffer (PBS) in 24-well plate containing RPMI 1640 medium at 5% CO_2_ and 37°C. The PBMCs were collected after 48 h of culture, by centrifugation and PrimeScript™ RT reagent kit (TaKaRa, CA, USA) was utilized to extract RNA from cells sediment as per the manufacturer’s guidelines. The cDNA-based quantifications of cytokine transcriptions were evaluated as previously stated protocol of RT-PCR. The reaction conditions and set of primers used for cytokines [interleukin-2 (IL-2), IL-10, TGF-β1, IL-6, IFN-γ, and IL-17] and endogenous reference gene (β-actin), as well as their amplification efficiencies are mentioned in Tables S1–S3 in Supplementary Material. The data were analyzed based on Raw cycle thresholds (Ct), obtained from the ABI Prism 7500 software (Applied Biosystems, USA) by comparative Ct (2^−ΔΔ^ Ct) method (26).

### Analysis of Major Histocompatibility Complex (MHC)-I and -II Molecule Expression

After separation of PBMCs by standard Ficoll-hypaque (GE Healthcare, USA) gradient centrifugation and two times washing with the PBS (Ca^2+^/Mg^2+^-free, pH 7.4), were poured into flat-bottom six well culture plates (Corning Inc., USA) containing RPMI 1640 medium (Invitrogen, USA) supplemented with 10% FBS and penicillin + streptomycin (Invitrogen, USA). The monocytes stick to the bottom of the plate, were collected (27) and adjusted at density of 1 × 10^6^ cells/ml, whereas, Non-sticky cells were discarded by multiple washing steps. The purified monocytes were incubated in 24-well culture plates with different concentrations of rHcSTP-1 (treatment group) or pET32a protein and PBS (control group) at 37°C for 24 h. Subsequently, the monocytes were marked with monoclonal antibodies MHC-I (MCA2189A647, AbDserotec, Bio-Rad, USA) and MHC-II (MCA2225PE, AbDserotec), followed by flow cytometric analysis at FACS Calibur cytometer (BD Biosciences, San Jose, CA, USA) (Figure 7A).

### Cell Proliferation Assay

The 100 µl of freshly isolated PBMCs at density of 1 × 10^6^ cells/ml was poured into 96-well culture plate and stimulated with ConA (10 µg/ml). The PBMCs were incubated with serial concentrations of rHcSTP-1 protein (10, 20, and 40 µg/ml) in treatment group and pET32a empty protein or control vehicle (PBS) were added into control groups at 5% humidity and 37°C temperature for 72 h. Detection of PBMC proliferation was carried out according to the previously mentioned procedure of cell proliferation assay kit (28). 10 µl of CCK-8 solution (Beyotime China) was mixed in every well of plate 6 h prior to harvest and absorbance were checked by using spectrophotometer (Bio-Rad, USA) at wavelength 450 nm (OD_450_). Result presented here are from three independent experiments.

### Cell Migration Assay

The migration activity of cultured PBMCs was measured with 8.0-µm pores Millicell^®^ insert (Merck-Millipore, USA) as stated previously. Briefly, in treatment group 200 µl of freshly separated cells were poured into the upper chamber with various concentrations of rHcSTP-1 (10, 20, and 40 µg/ml) and pET32a control protein or equal volume of control buffer (PBS) were added in control groups. Subsequently, 1,300 µl cell culture medium was filled in the lower chamber for 2 h incubation at 37°C and 5% CO_2_. After that, columns were removed, and PBMC passed through polycarbonate layer were figure out by a Neubauer counting chamber. Data were resulted as percentage of seeded PBMCs. Three independent experiments were conducted.

### Intracellular Nitrite Production by PBMCs

The goat PBMCs were separated and washed with Ca^2+^/Mg^2+^-free PBS (pH 7.4). 100 µl of cells containing DMEM medium was poured in 96-well plate with varying concentrations of rHcSTP-1 (10, 20, and 40 µg/ml), similarly the control groups were incubated with recombinant protein of pET32a or PBS (control buffer). NO production by cells was determined by Griess assay (29), by using Total Nitric Oxide Assay Kit (Beyotime, China) according to the manufacturer’s instructions. The values in the reaction mixture were measured at microplate spectrophotometer (Bio-Rad Laboratories, USA) at 540 nm (OD_540_) wavelength. Data presented here are results of triplicate experiments.

### Apoptotic Efficiency of rHcSTP-1 on PBMC

Efficiency of rHcSTP-1 to induce apoptosis of PBMC was assessed by flow cytometric analysis (BD Biosciences, USA). The PBMCs (1 × 10^7^ cells/ml) treated with different rHcSTP-1 protein concentrations (5, 10, 20, and 40 µg/ml) and empty pET32a protein, were stained with Annexin V-FITC kit (Miltenyi Biotec, Germany) according to the manufacturer’s protocols. The apoptosis rate was calculated by the percentage of early (Annexin V^+^ PI−) and late (Annexin V^+^ PI^+^) PBMC apoptosis. Cells without any treatment were set as control. Data were analyzed using Flow Jo 7.6 software (Tree Star, USA).

### Statistical Analysis

Data analysis regarding all experiments was performed by using the statistical package, GraphPad Premier 6.0 (GraphPad Prism, San Diego, CA, USA). Data are presented as mean ± SEM. The differences between groups were compared by one-way ANOVA, followed by a Tukey test and were considered statistically significant at *p* < 0.05.

## Results

### Cloning and Protein Expression of *H. contortus* STP-1

**Figure 1:**
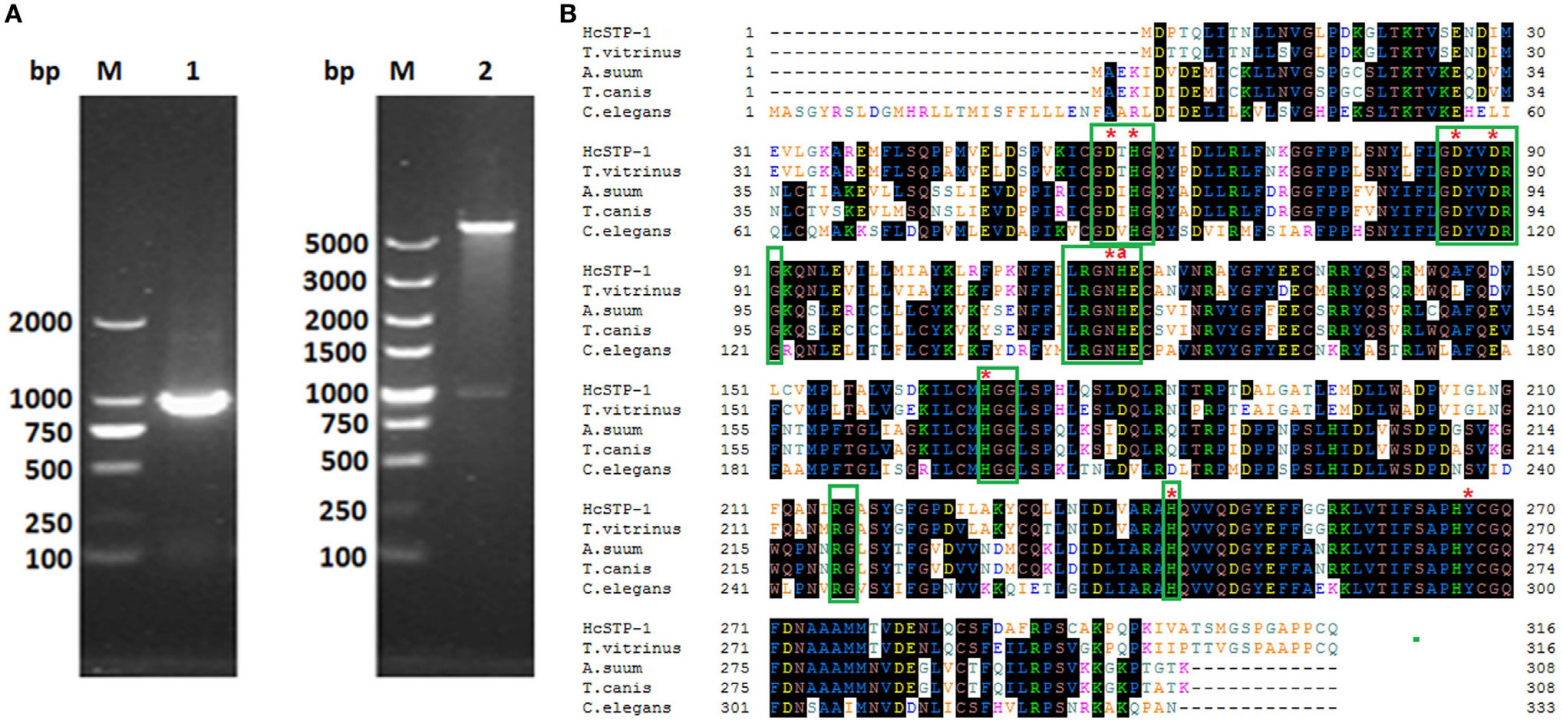
Cloning and sequence analysis of HcSTP-1 gene. **(A)** Electrophoresis of PCR product of HcSTP-1 open reading frame (Lane 1). Restriction enzyme digestion of pET32a-HcSTP-1 with *BamH* I and *Xho* I (Lane 2) and molecular weight DNA marker (Lane M). **(B)** The comparison of amino acid sequence of *Haemonchus contortus* serine/threonine-protein phosphatase 1 (STP-1) and with predicted STPs from *Trichostrongylus vitrinus* (CAM84506.1), *A. suum* (CAJ98743.1), *Toxocara canis* (KHN86897.1), and *C. elegans* (NP_505086.2). Amino acids predicted to be involved in the catalytic metal binding are indicated by asterisks 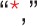 and the histidine residue proposed for proton donation is indicated with 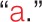 The conserved motifs found in all sequences including Prosite motif PS00125 (LRGNHE) are boxed in green color.

The RT-PCR generated fragment of STP-1 gene with molecular size of 951 bp, was obtained from *H. contortus* cDNA and successfully cloned into an expression vector system, confirmed by enzymatic digestion with *BamH* I and *Xho* I (Figure 1). After that, cloned HcSTP-1 sequence was translated into 316 amino acids with predicted 35 kDa molecular mass and calculated pI of 6.328. The nucleotide and amino acid sequence identity HcSTP-1 using BLASTx and BLASTp revealed highly sequence resemblance with STPs from other parasite species, existing at NCBI database (see text footnote 1). In this study, characterization of HcSTP-1 cDNA showed 98% significant sequence identity to *H. contortus* (ADJ96628.1), 80% with *C. briggsae* (XM_002631635.1) (data not shown). Multiple sequence alignment showed that HcSTP-1 polypeptide shares a significant similarity of 91% with *T*. *vitrinus* (GenBank: CAM84506.1), 62% with *T*. *canis* (GenBank: KHN86897.1), 62% with *Ascaris suum* (GenBank: CAJ98743.1), and 58% with *Caenorhabditis elegans* (GenBank: NP_505086.2). The BLAST analysis predicted number of conserved metal ion-binding sites and histidine residue predicted to be involved in proton donation. The amino acids expressing STP protein motif LRGNHE was found during sequence analysis. The aligned STPs sequences were highly similar in central regions, particularly in term of characteristics sequence motifs, such as GDXHG, GDYVDRG, GNHE, HGG, RG, and H (30), whereas there was a higher variation occurred regarding to N- and C-terminal regions among all align sequences (Figure 1). Further sequence analysis indicated lack of signal peptide, transmembrane domain, and GPI anchor site, whereas T and B cell motifs were predicted in deduced protein structure (Figures S1–S4 in Supplementary Material).

**Figure 2:**
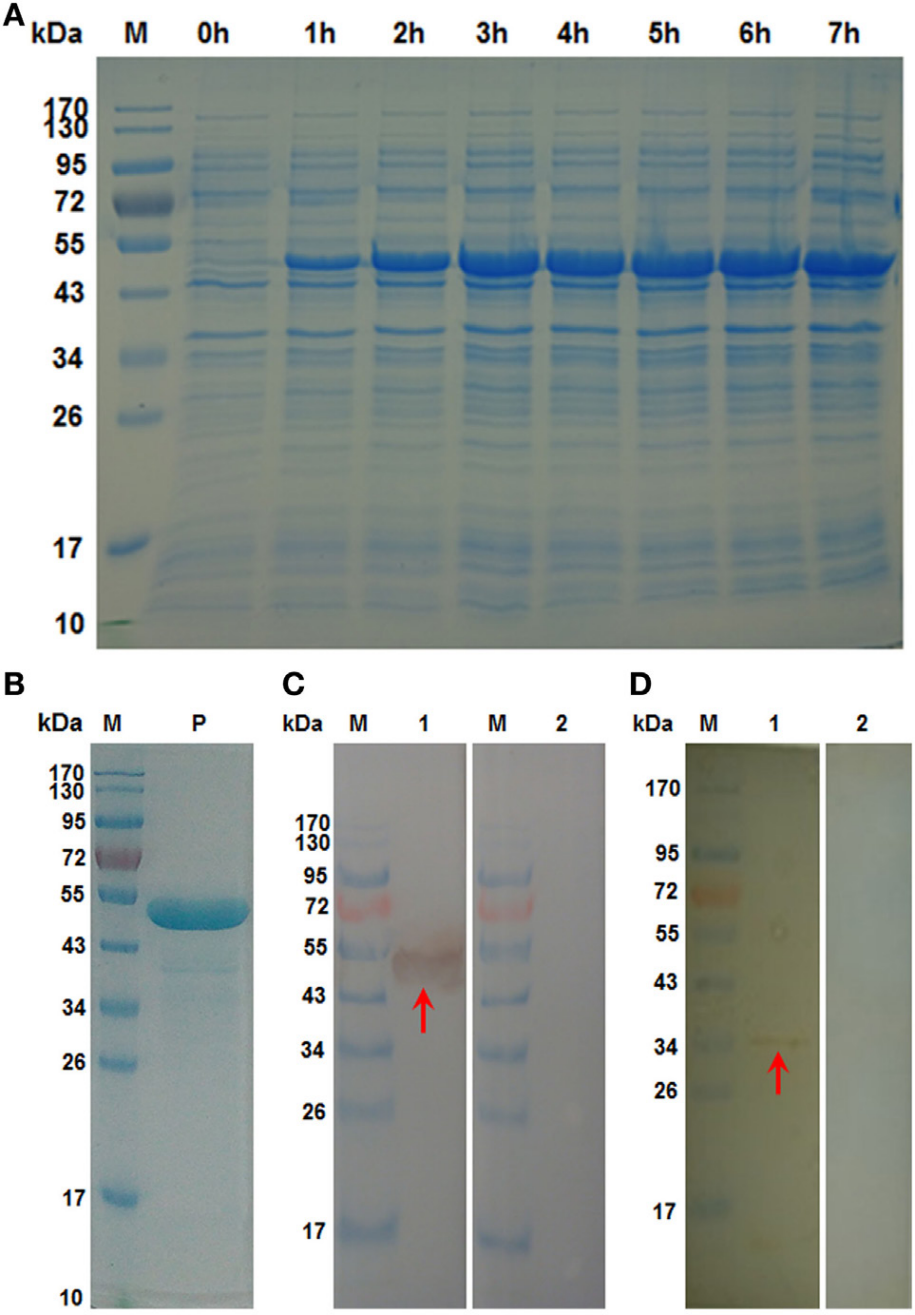
Expression, purification and immuno reactivity of rHcSTP-1. **(A)** Recombinant expression vector before (Lane 0 h) and after 1 mM IPTG induction (Lane 1–7 h). Lane M: standard protein molecular weight marker. **(B)** Purified rHcSTP-1 protein resolved on sodium dodecyl sulfate-polyacrylamide gel electrophoresis (Lane P). **(C)** Western blot of rHcSTP-1 protein. (Lane 1) Purified recombinant protein was electrophoresed and transferred to a membrane probed with serum from goat infected with *Haemonchus contortus* and (Lane 2) with normal goat serum as negative control. **(D)** Western blot of total ES proteins of *H. contortus*. (Lane 1) ES proteins from *H. contortus* parasites were detected by rat antibodies against serine/threonine-protein phosphatase 1 (STP-1) protein and (Lane 2) with serum from normal rat as control.

The recombinant product of HcSTP-1 gene inserted into *E. coli* was expressed after IPTG induction and detected after staining with Coomassie brilliant blue R-250 on SDS-PAGE. The rHcSTP-1 protein was expressed with molecular weight of about 53 kDa (Figure 2, Lane P) along with 18 kDa calculated mass of vector protein (pET32a). The target protein was confirmed at its original size of 35 kDa after reduction from vector protein. Polyclonal antibodies against rHcSTP-1 were identified by immuno-blot analysis, which revealed that rHcSTP-1 could be recognized by sera from goats infected with *H. contortus* and native STP-1 protein from *H. contortus* could be recognized by antibodies generated against rHcSTP-1 protein (Figures 2C,D, Lane 1). However, no protein was detected with normal sera taken from un-immunized goats/rats (Figures 2C,D, Lane 2).

### HcSTP-1 Localized Outer and Inner Lining of *H. contortus* Worm Sections

**Figure 3:**
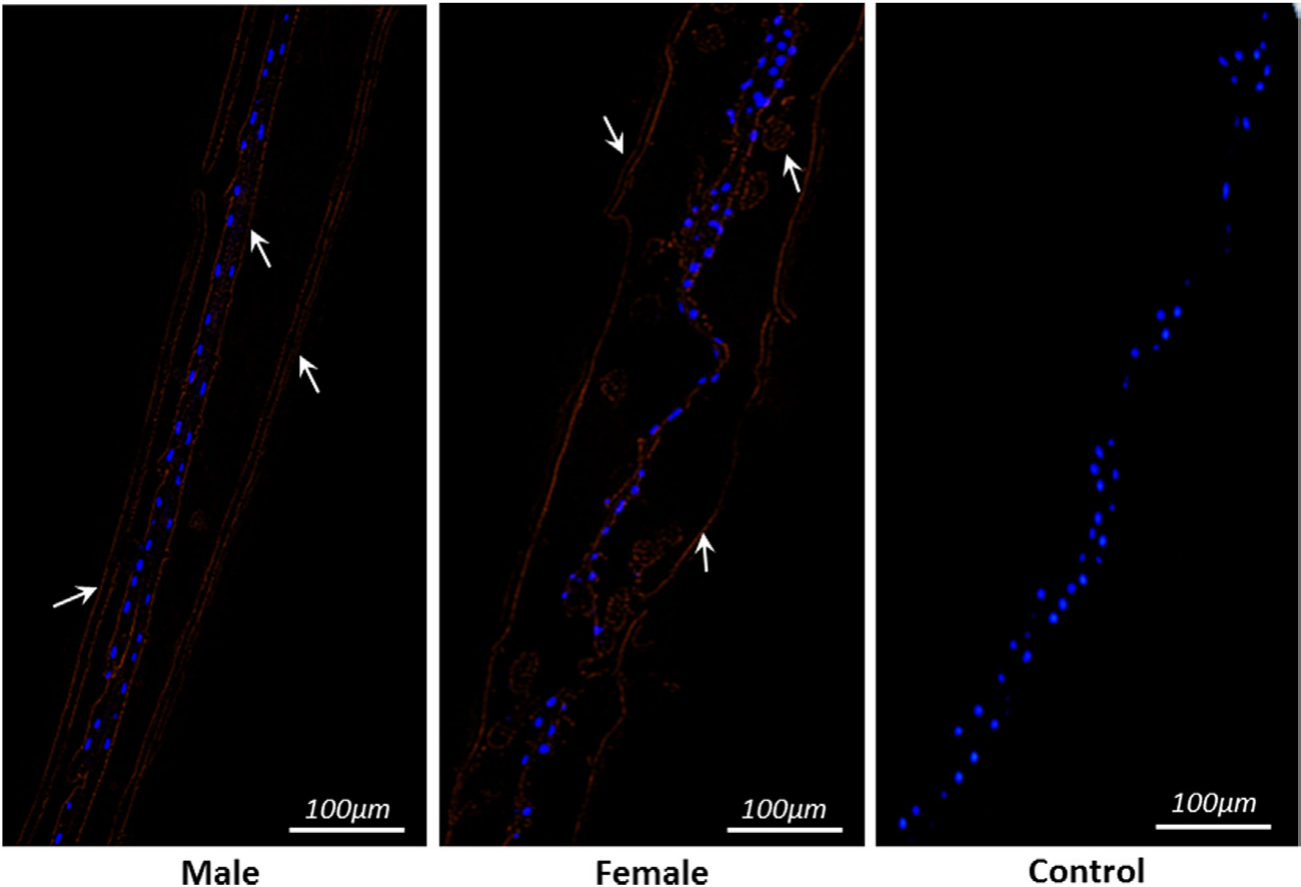
Localization of serine/threonine-protein phosphatase 1 protein within parasite structure. Immunohistochemically STP-1 protein localizes inner and outer structure of *Haemonchus contortus* male and female adult worms. The red color indicates target protein stained with Cy3 and blue dots are localization of nuclei stained with DAPI. No red fluorescence was detected in control panel. Scale-bars: 100 µm.

The partial body sections of male and female adult worms are shown in Figure 3. Immunohistochemical analysis was performed to detect distribution of native STP-1 protein within the worms. The red lining at outer and inner layer of membranes and in gut region strongly indicated the presence of this protein within worms sections. However, no fluorescence was detected in the control section (Figure 3).

### rHcSTP-1 Binding Confirmation on Surface of Goat PBMCs

**Figure 4:**
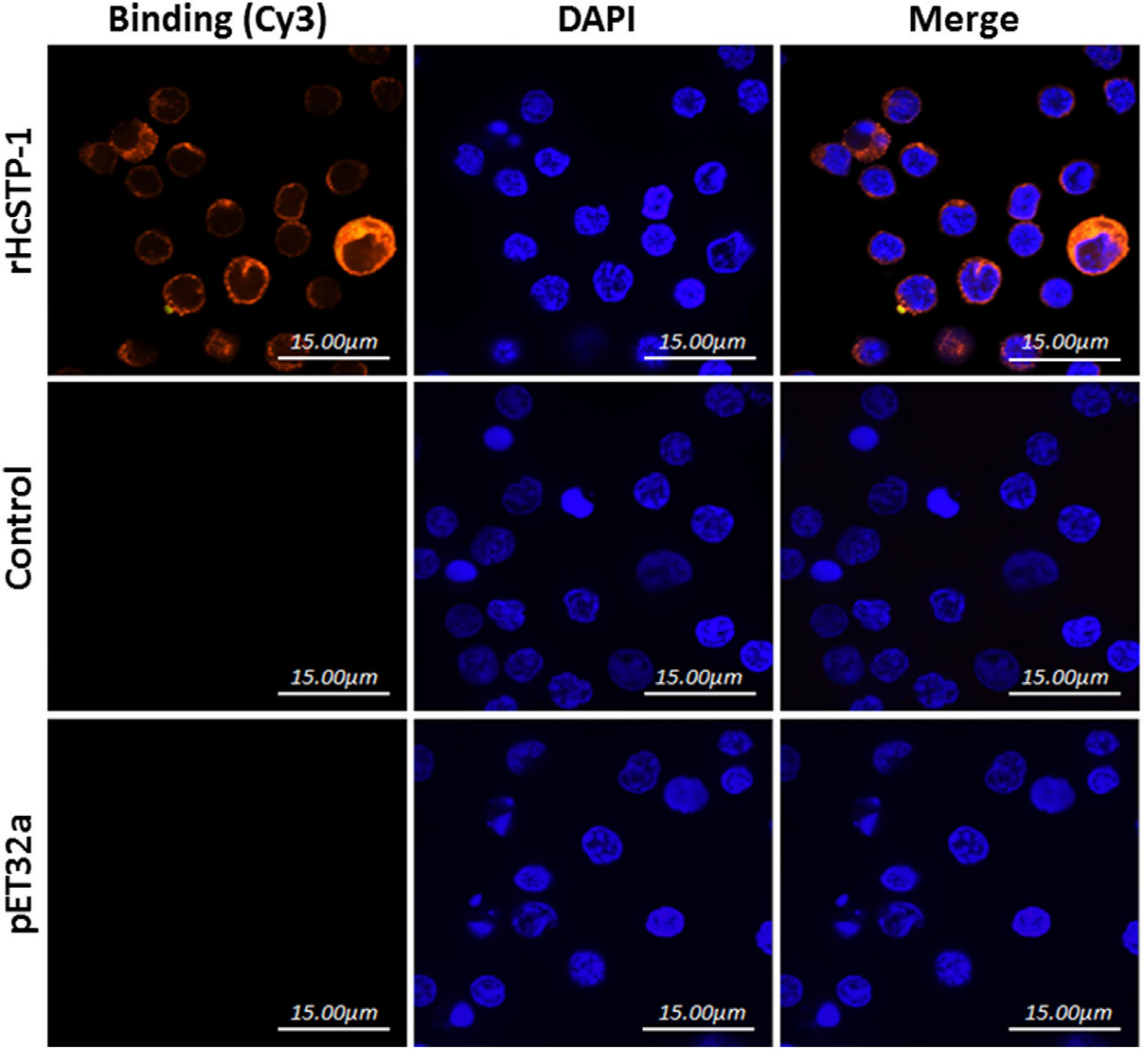
Binding of rHcSTP-1 protein with goat peripheral blood mononuclear cells *in vitro*. The cells were incubated with rat sera anti-rHcSTP-1-O IgG, anti-HisTag protein (pET32a) or negative rat IgG as primary antibody followed by Cy3-labeled goat anti-rat IgG (red) as secondary antibody. Red fluorescence on surface of cells showed target protein staining (Cy3) and nuclei of cells were visualized by DAPI (blue). No red fluorescence was observed in control groups. The results were analyzed by using confocal laser scanning microscopy.

The interaction of HcSTP-1 with PBMCs, which are key elements for immune responses, was investigated by using IFA. As shown in Figure 4, the Cy3-labeled target protein and the DAPI labeled nuclei displayed red and blue fluorescence, respectively. The red visualization on the surface of cells in treatment group (rHcSTP-1) indicated the binding of the protein whereas, no red labeling was detected in the control groups (pET32a or PBS), which clearly specified that rHcSTP-1 could bind on the surface of goat PBMCs and exerts its immune effects in a multiple manners (Figure 4).

### rHcSTP-1 Decreased Anti-Inflammatory and Increased Pro-Inflammatory Cytokines Expression in a Balanced Th1-Th2 State *In Vitro*

**Figure 5:**
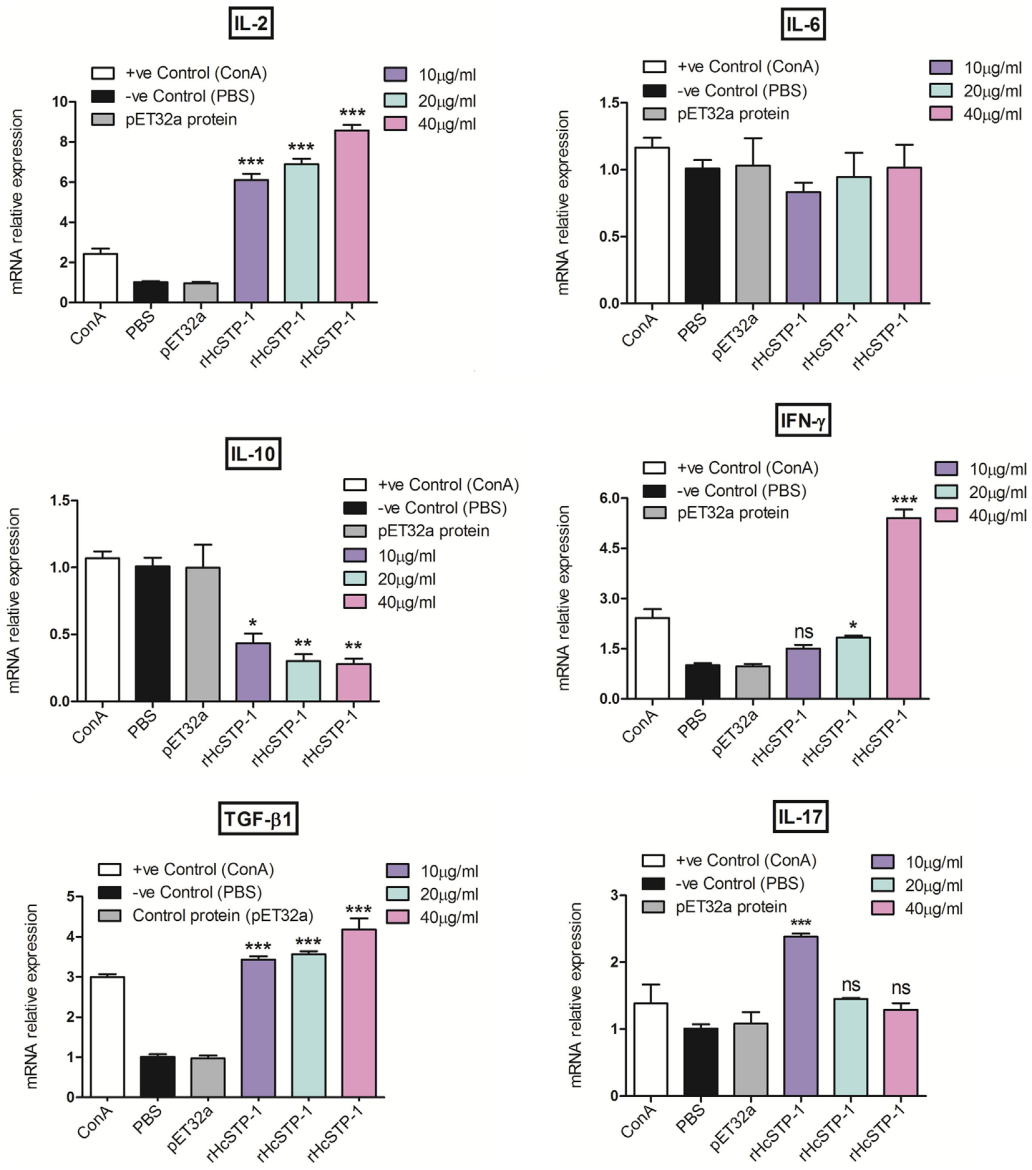
Relative expression of multiple cytokines in goat peripheral blood mononuclear cells stimulated by the recombinant HcSTP-1. Cells were incubated with the recombinant serine/threonine-protein phosphatase 1 (STP-1) for 48 h, the mRNAs encoding interleukin-2 (IL-2), IL-6, IL-10, IFN-γ, TGF-β1, and IL-17 were quantified by reverse-transcription-polymerase chain reaction. The significant level was set at *p < 0.05, **p < 0.01, ***p < 0.001, and ns non-significant compared with the untreated group (control). Data are representative of three independent experiments.

The mRNA levels of cytokines produced by cultured goat immune cells (PBMCs) in response to STP-1 protein were tested at RT-PCR machine. As depicted in results of Figure 5, transcriptions level of IL-2 [ANOVA, *F*_(5,12)_ = 198.9, *p* = 0.0001] and transforming growth factor-β1 (TGF-β1) [ANOVA, *F*_(5,12)_ = 108.0, *p* = 0.0001] were increased significantly at dose-dependent manner to that of pET32a protein and PBS control group. However, the IL-10 transcription was decreased [ANOVA, *F*_(5,12)_ = 19.04, *p* = 0.0001] dose dependently to that of control groups. Here, we also noted that interferon gamma (IFN-γ) at protein concentration of 10 µg/ml showed no difference (*p* = 0.337); however, at 20 µg/ml (*p* = 0.002) and 40 µg/ml [ANOVA, *F*_(5,12)_ = 105.6, *p* = 0.0001] the transcription level was augmented significantly. After incubation with rHcSTP-1 protein, the expressions of IL-17 at 10 µg/ml reached at its significant level [ANOVA, *F*_(5,12)_ = 11.76, *p* = 0.0003], while at 20 and 40 µg/ml remained non-significant. However, IL-6 was not significantly different [ANOVA, *F*_(5,12)_ = 0.604, *p* = 0.699] when compared with control groups (Figure 5).

### rHcSTP-1 Protein Affected PBMCs Proliferation

**Figure 6:**
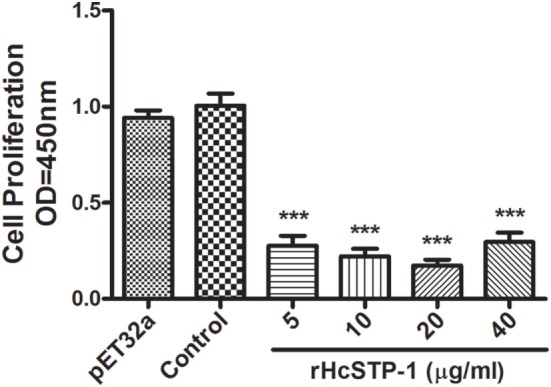
Influences of different concentrations of rHcSTP-1 on peripheral blood mononuclear cells proliferation *in vitro*. Cells were simulated with ConA and treated with control buffer, His-Tag protein (pET32a), and various concentrations of recombinant serine/threonine-protein phosphatase 1 (STP-1) protein for 72 h. Proliferation test was conducted by CCK-8 and OD450 value was measured using microplate spectrophotometer. Cell proliferation index was calculated by considering the OD450 values in controls as 100%. The data are expressed as mean ± SEM of three independent experiments (***p < 0.001).

Antiproliferative effects of rHcSTP-1, compared to the negative control and control protein treated groups were evaluated by using cell counting kit (CCK8). The results demonstrated that, cells stimulated with ConA and incubated with rHcSTP-1 protein downregulated [ANOVA, *F*_(5,12)_ = 67.38, *p* = 0.0001] the multiplication efficiency dose dependently (Figure 6). However, no significant change was observed between control groups [ANOVA, *F*_(5,12)_ = 67.38, *p* = 0.557].

### rHcSTP-1 Inhibited MHC-I and -II Expression on Goat Monocytes

**Figure 7:**
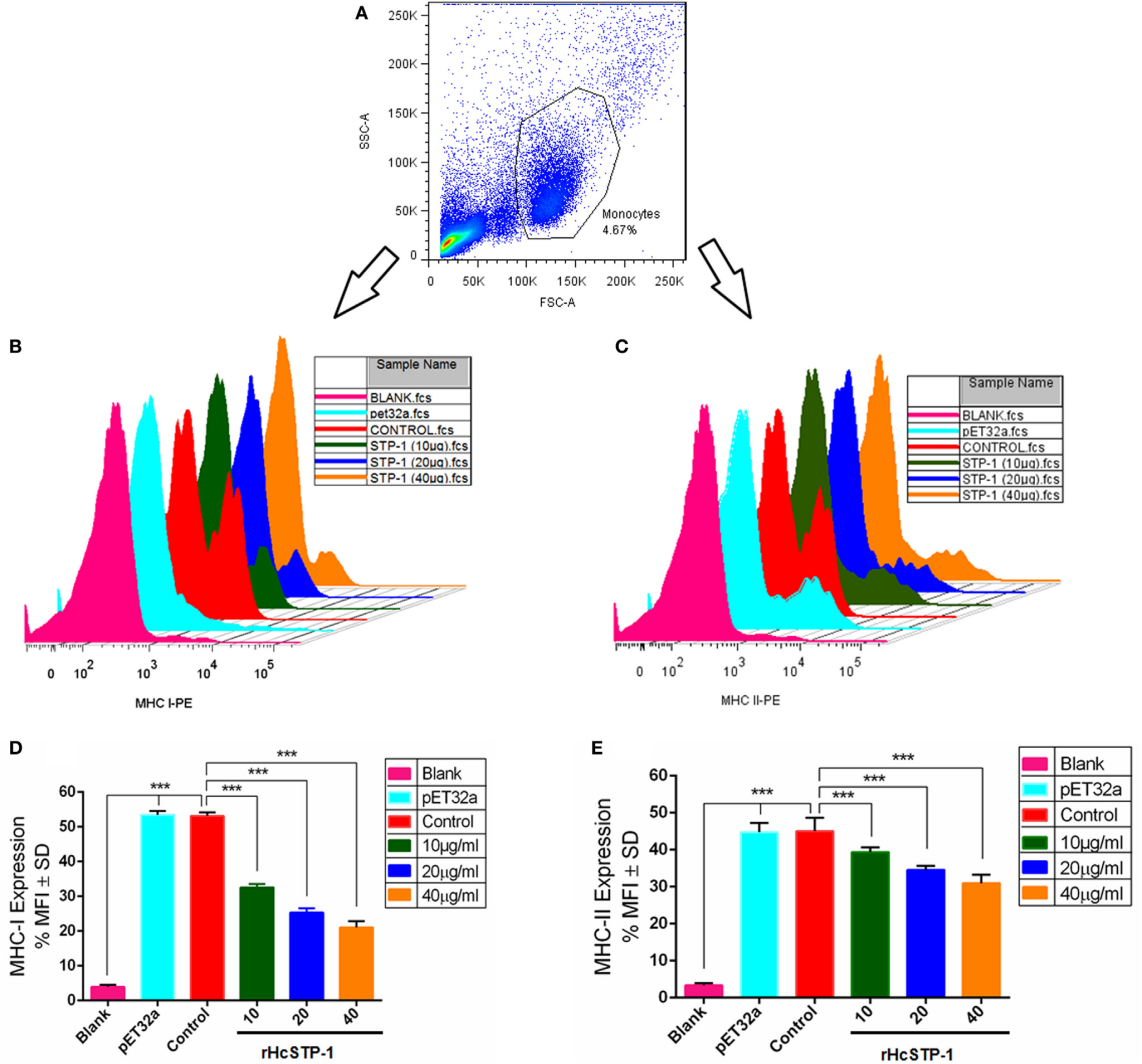
rHcSTP-1 suppressed both major histocompatibility complex (MHC) class-I and II on goat monocytes. Monocytes were cultured in the presence of control buffer, HisTag protein (pET32a), and multiple concentrations of rHcSTP-1 for 24 h. MHC-II expression was measured by flow cytometric analysis and calculated as the percentage of mean fluorescence intensity (MFI). **(A)** Histogram corresponds to one representative of three independent experiments. **(B,C)** Stagger offset showing expression level of MHCs on monocytes. **(D,E)** Bar graphs represent the MFI ± SD of controls. The data are representative of three independent experiments (***p < 0.001).

In response to the rHcSTP-1, the expression of MHC-I and -II surface markers on monocytes were evaluated, and results showed that as compared to expression in control groups, rHcSTP-1 significantly decreased MHC-I (Figures 7B,D) molecule expression [*F*_(5,12)_ = 780.3] as well as MHC-II [*F*_(5,12)_ = 157.2] on the surface of the goat monocytes in a dose-dependent manner *p* < 0.0001 (Figures 7C,E).

### PBMC Migration Assay

**Figure 8:**
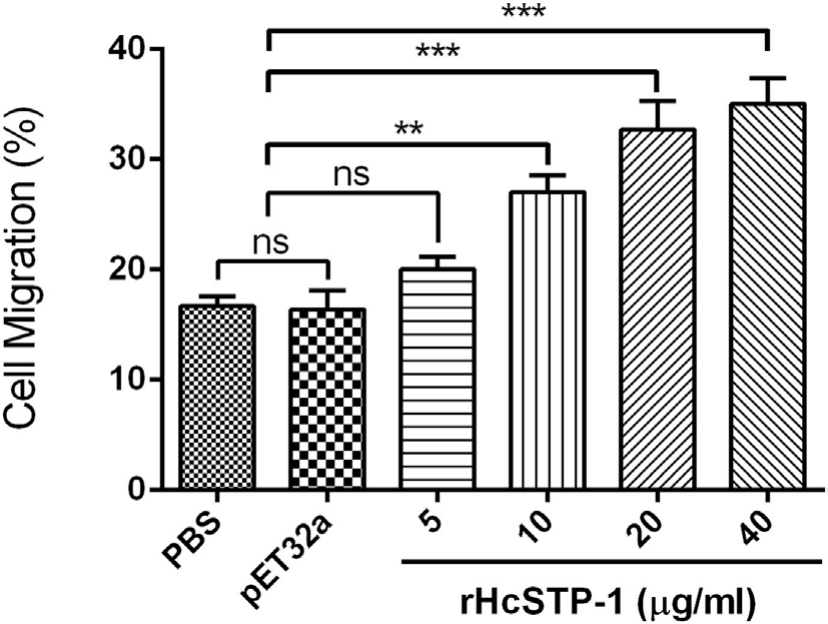
Effects of the various concentrations of rHcSTP-1 on peripheral blood mononuclear cell migration *in vitro*. Cells were treated with control buffer, HisTag protein (pET32a), and multiple concentrations of rHcSTP-1. The migration percentage was determined randomly. The difference between the mean values was calculated using ANOVA. Data are representative of three independent experiments (**p < 0.01, ***p < 0.001, and ns non-significant versus the control).

To investigate impact of the rHcSTP-1 on immune cell migration, the percentage of migrated cells through Millipore polycarbonate membrane into the lower chamber was calculated. Results showed that 16.67 ± 0.8819% cells with control and 16.33 ± 1.764% cells with control protein group (pET32a) were migrated into lower chamber (*p* = 0.087). At 10 µg/ml (27.00 ± 1.528%), 20 µg/ml (32.67 ± 2.603%), and 40 µg/ml (35.00 ± 2.309%) rHcSTP-1 protein concentrations, significant amount of cells were passed through membrane (Figure 8). However, no difference was found between control and 5 µg/ml (20.00 ± 1.155%) protein concentration (*p* = 0.084).

### rHcSTP-1 Involved in Intra-PBMCs NO Production

**Figure 9:**
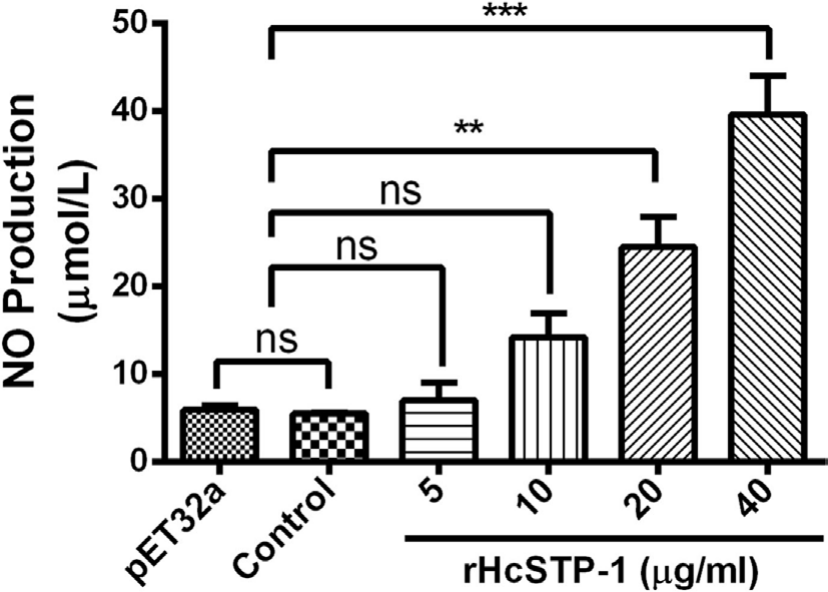
Measurement of the intracellular nitric oxide production by goat peripheral blood mononuclear cells *in vitro*. Nitric oxide concentration in the supernatant of cell cultures was performed according to the instructions of Griess assay by Total Nitric Oxide Assay Kit. The data were presented here as mean ± SEM of three independent experiments (**p < 0.01 and ***p < 0.001).

Nitric oxide plays a crucial role in the host defense mechanism by preventing growth or killing the parasite directly during infection. By using total nitric oxide kit, we found that no significant production was occurred [ANOVA, *F*_(5,12)_ = 25.92, *p* = 0.504] at 5 and 10 µg/ml compared with the groups treated as control. Here, we also observed that at 20 and 40 µg/ml, a significant level of NO was produced [ANOVA, *F*_(5,12)_ = 25.92, *p* = 0.0001]. However, production of NO between control (PBS) and pET32a group was also non-significant [ANOVA, *F*_(5,12)_ = 25.92, *p* = 0.473] (Figure 9).

### rHcSTP-1 Dramatically Modulated Early and Late Apoptosis of PBMCs

**Figure 10:**
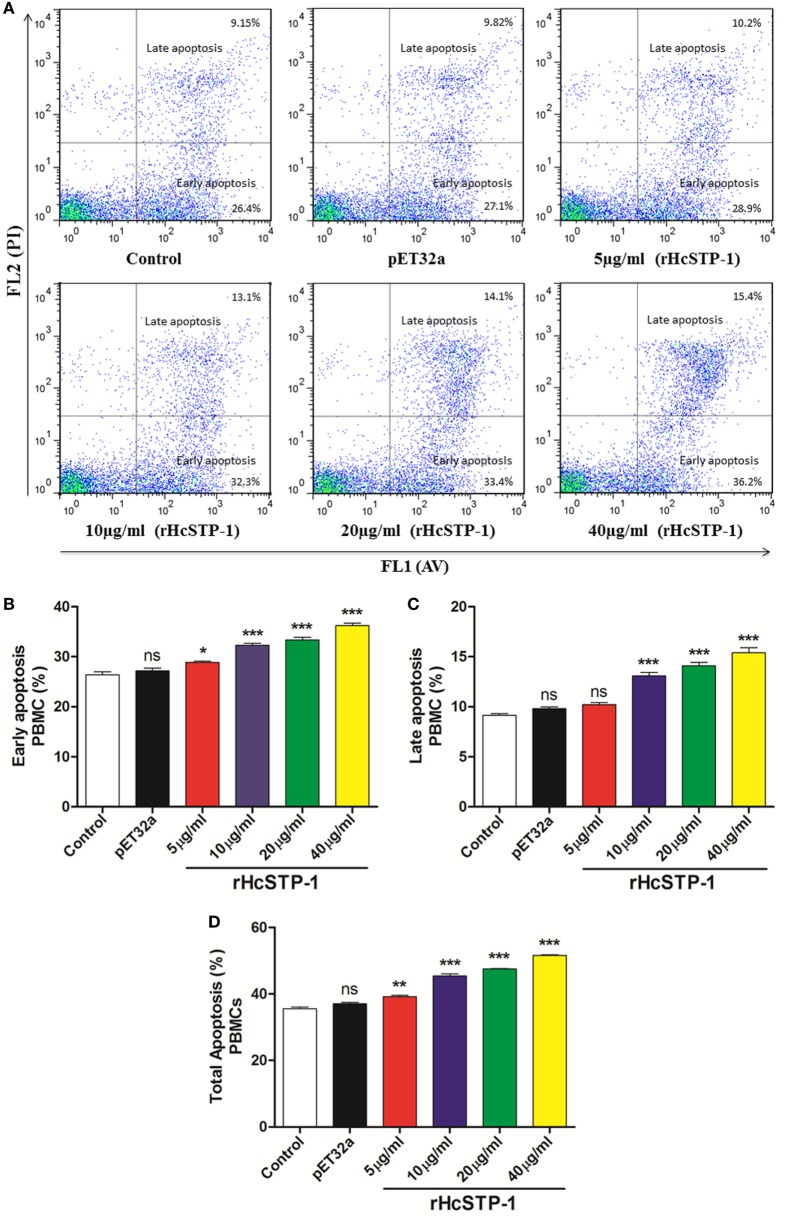
Apoptotic analysis of peripheral blood mononuclear cells (PBMCs) by flow cytometry. **(A)** The dot plot showing percentages of PBMCs undergoing early and late stage apoptosis dually stained with Annexin V-FITC/PI. **(B)** Bar graph showing percentage of early stage apoptosis of PBMCs after rHcSTP-1 treatment. **(C)** Bar graph showing percentage of late stage apoptosis of PBMCs after rHcSTP-1 treatment. **(D)** The effect of the different protein treatments on total % (early + late) of apoptotic cells. Data are presented as the mean ± SEM (*n* = 3) from triplicate experiments. Asterisks designate treatment groups are significantly differ in respect to control one (*p < 0.05, **p < 0.01, ***p < 0.001, and ns non-significant).

It has been suggested that STPs obviously involved in various cellular processes including cell viability and cell death (10). The apoptotic activity of rHcSTP-1 on PBMCs was evaluated by using the externalization of membrane phospholipid phosphatidylserine (PS) as a marker of cell apoptosis. Flow cytometry assay revealed that cells incubated with different protein concentrations (5, 10, 20, and 40 µg/ml) showed significantly increased frequency of apoptotic percentage in both early [ANOVA, *F*_(5,12)_ = 69.08, *p* = 0.0001] and late stage apoptosis [ANOVA, *F*_(5,12)_ = 68.30, *p* = 0.0001] compared with the control groups. Meanwhile, no significant change was observed [ANOVA, *F*_(5,12)_ = 200.8, *p* = 0.0986] between control and pET32a group (Figure 10).

## Discussion

Identification and development of novel drug targets are always a primacy in the biological study of socio-economically important parasites. Helminths ESPs including some antigens with unknown biological functions were characterized (31–33), and due to their sequences homology, these molecules have been proven as promising vaccine candidate against nematodes infections (34, 35). Among PPs, PP1 elaborate numerous biological activities, such as membrane receptors/channels regulations, protein synthesis, glycogen metabolism, transcription, cellular division, and apoptosis (36). In current investigation, an ES protein of *H. contortus* (STP-1) was characterized and localization of this protein through IFA confirmed its attachment with host immune cells for the first time, in shape of surface ligand complex (37), which is characteristic feature of ES proteins to suppress or modulate the immune functions of host PBMCs.

The PP1 is a major ubiquitously expressed STP, which shares a conserved catalytic domain with all other isotypes (PP2A, PP2B, PP4, PP5, PP6, and PP7) (38) and provides extensive information regarding their functions involved in numerous vital cellular mechanisms in all organisms (30, 39). In this study, highly significant amino acid identity of HcSTP-1 with presence of a conserved motif (LRGNHE) for STP protein pattern at positions 115–120 (18), involvement of amino acids for catalytic activity like metal ion-binding and proton donation were found (7). In the central regions of polypeptides, the amino acid sequences were highly conserved, particularly with the signature sequence motifs of the PPP family (GDXHG, GDXVDRG, GNHE, HGG, RG, and H) (30). However, the N- and C-terminal amino acids were variable, which suggested that it might be involved in protein binding and regulatory activity.

Previous studies suggested that STPs were associated with male reproductive processes of parasites, including sperm maturation, motility, and viability (40). In our previous proteomic analysis, STP-1 was found at adult and late adult developmental stages of *H. contortus in vivo* (19). In this study, immunohistochemically HcSTP-1 was localized in both male and female adult worms of *H. contortus*. Somehow, most PPs considered to be conserved during free-living as well as parasitic developmental stages; however, the biological pathways involved in the growth, development, survival, and/or reproduction reflects between invertebrates and vertebrates. Based on current information, a deep insight into the biochemical localization of STPs in *H. contortus* is required to understand the gender dependent immune stimulation by STPs during host–parasite interface.

The immune (Th1 and Th2) and inflammatory responses are deeply allied with secretion of various chemokines and cytokines by number of immune cell types (lymphocytes and monocytes), which perform crucial immunoregulatory functions (41), against parasitic infections like *H. contortus* (42). In this study, the levels of mRNA transcripts for IL-2, interleukin-10, transforming growth factor beta 1, interleukin-6, interferon-γ, and interleukin-17 in cultured PBMCs after treatment with rHcSTP-1 were detected for the first time. Schallig (43) suggested that sheep challenged with *H. contortus* infection responded cell mediate immune reaction (Th1), categorized by upregulation of IFN-γ and IL-2 mRNA expression. Similarly, in current research, goat PBMCs in response to rHcSTP-1 protein showed higher expression levels of IL-2 and IFN-γ, suggesting that these cytokines might be in favor of non-protective Th responses against hemonchosis. The immunosuppressive cytokine IL-10 was responsible for inhibition of Th2 immune responses (44, 45). In this research, rHcSTP-1 protein showed a significant Th2 type immune response by decreased IL-10 cytokine in culture PBMCs, and thus exerts suppressive potential on differentiation of T_reg_ cells, which might be immune modulatory role of HcSTP-1 against parasite infection.

Previously, some researches demonstrated that IL-17 cytokine were involved in pathogenesis and inflammatory responses by numerous parasites (46–48). It was also elucidated that decreased level of IL-17 resulted favorable host protective responses (49). In our previous study, HcESPs were found to induce IL-17 cytokine by stimulation of the Th17 cells and resulted in inflammatory reaction favorable for worm survival in host. In this study, we found that rHcSTP-1 with concentration of 10 µg/ml significantly upregulated IL-17 cytokine. Our findings indicated that HcSTP-1 protein of *H. contortus* was adept in Th17 cells induction, which lead to inflammatory response. However, the resulted inflammatory reactions might be in favor of parasitic infection. TGF-β1 is functionally multidimensional cytokine that controls various immune system activities differentially on different cell types and potentially regulate various biological processes (50). It also plays vital roles to inhibit proliferation and to stimulate the apoptosis of various immune cells subset (51). In this investigation, the binding of recombinant protein HcSTP-1 to the goat PBMCs increased the production of TGF-β1, which could be a one of mechanism of rHcSTP-1 protein to facilitate immune evasion during host–parasite interactions.

Major histocompatibility complex is associated with many important immunological activities like: stimulation of antibody production and immune responses against microbial and parasitic pathogens (52, 53), although some cytokines especially IFN-γ and TGF-β (54) have been proven to upregulate MHC-I and -II gene expression in different immune cells. Here, we noted that STP-1 protein of *H. contortus* potentially suppress the MHC-I and -II molecules expression in goat monocytes. This indicated that STP-1 protein interferes with the MHC-I and -II antigen presentation pathway, which might be a key mechanism for evasion from the host’s immune response.

The cellular processes such as cell migration part play an importance role in immune surveillance and tissue regeneration, by recruiting of immune cells into the surrounding tissue to destroy invading microorganisms. In addition, Type-1 as precise inhibitor of protein serine/threonine phosphatase (PHI-1) participated in regulatory events by modulating the migration of human endothelial and epithelial cells (55). Moreover, it was suggested that helminths actively induce migration of immune cell to infected sites (56). In this research, migration experiment concluded that rHcSTP-1 is an important binding protein to the goat PBMCs, which regulate the migration of immune cells to combat with invading *H. contortus* parasite and might be a contributor of HcESPs in the PBMCs migration.

Many proteins including ser/thr phosphatases were thought to be implicated in regulation of apoptosis (57). Till now, studies on cell death regulation by PPP subfamilies like PP2A and PP2B were well documented (10, 58); however, few studies highlighted the direct association and involvement of PP1, PP4, PP5, PP6, and PP7 members of phosphatase family in apoptosis (59–62). This study showed that rHcSTP-1 was involved in apoptosis as well as cell necrotic activity at dose-dependent manner.

In addition, cell proliferation is an indispensable process which is regulated or suppressed by both antigen-presenting cells (APCs) and T cells, thus modulate the host immune responses (63). In our earlier work, HcESPs significantly suppressed PBMCs multiplication at dose-dependent manner. In this study, PBMCs proliferation was decreased at significant level, which ultimately increased the pre- and post-apoptotic percentage in response to rHcSTP-1 treatment. Our study demonstrated that rHcSTP-1 as a constituent of HcESPs might interfere with modulatory activity on PBMCs proliferation and cell death regulation; however, the exact mechanism and pathways involved during host–parasite interaction upon HcSTP-1 protein involvement remains to be determined.

The immune cell-derived molecule, nitric oxide (NO) has been concerned with host non-specific defense against variety of infections such as hemonchosis, leishmaniasis, trypanosomiasis, malaria, toxoplasmosis, and schistosomiasis (64, 65). Previous studies provoked that PPs including P1 and P2A are involved in iNOS assertion in murine macrophages (66). Another study also witnessed that serine-threonine phosphatases post-transcriptionally regulate iNOS translation in rat hepatocytes (67). Furthermore, IFN-γ and TNF-α activated release of NO by macrophages was usually associated with killing functions on the helminths (68). In our previous study, the generation of NO was significantly suppressed by HcESPs due to decreased level of IFN-γ (69). In this study, intracellular nitrite production of goat PBMCs was significantly upregulated in a concentration-dependent fashion. These findings suggested that *H. contortus* STP-1 protein could induce the production of NO through increasing IFN-γ level which might mediate immunomodulatory functions on NO production and thus intensified the harmful effects of some chemical factors produced by the host cells on the helminths during host–parasite interface.

## Conclusion

The current observations highlighted that *H. contortus* STP-1 as an important constituents of HcESPs localized in both gender, play pivotal stage-specific regulatory roles in interactions with host immune cells. This protein after being bind to host immune cells, regulate cytokines expression, cell proliferation, MHC expression, cell migration, NO production, and cell death regulation differentially. This study might be a powerful clue and would have major fundamental significant contribution in the discovery of novel therapeutic approaches to control hemonchosis. However, immune cells specific role of rHcSTP-1 and other related phosphatases subunits with PBMC sub-populations like T cell and B cell, NK cell, APCs, and macrophages involved in host–parasite relationship in activating immune and cellular response are worthy for future research.

## Ethics Statement

All experiments were conducted in accordance with the guidelines of the Animal Ethics Committee, Nanjing Agricultural University, China. All experimental protocols were approved by the science and Technology Agency of Jiangsu Province. The approval ID is SYXK (SU) 2010-0005.

## Author Contributions

XLi concerned with project designing, management, and coordination of this study. ME analyzed data and wrote the manuscript. WW, JG, ML, and YW provided technical support during experiments. MWH, MH, and XLiu participated in data analysis. LX, RY, and XS helped for providing all necessary materials during experiments.

## Conflict of Interest Statement

The authors declare that the research was conducted in the absence of any commercial or financial relationships that could be construed as a potential conflict of interest.

## Footnotes

^1^https://blast.ncbi.nlm.nih.gov/Blast.cgi.
