# Supplementary figures and images for: The Serine/Threonine-Protein Phosphatase 1 From Haemonchus contortus Is Actively Involved in Suppressive Regulatory Roles on Immune Functions of Goat Peripheral Blood Mononuclear Cells
Source: Front Immunol. 2018 Jul 16;9:1627. doi: 10.3389/fimmu.2018.01627 (PMC6054924; doi:10.3389/fimmu.2018.01627)

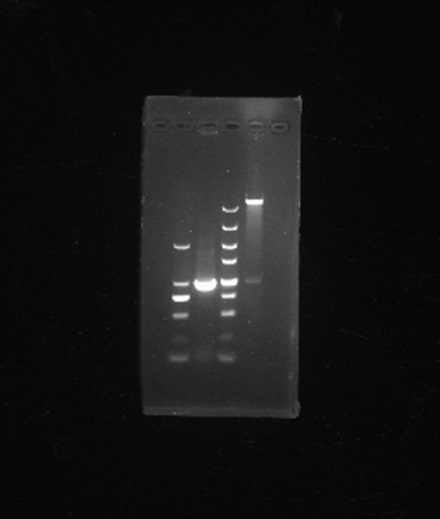

Supplement: Supplementary file 1 [file Data_Sheet_1.zip › new figs/Raw Image of Figure 1-Panel A.tif]

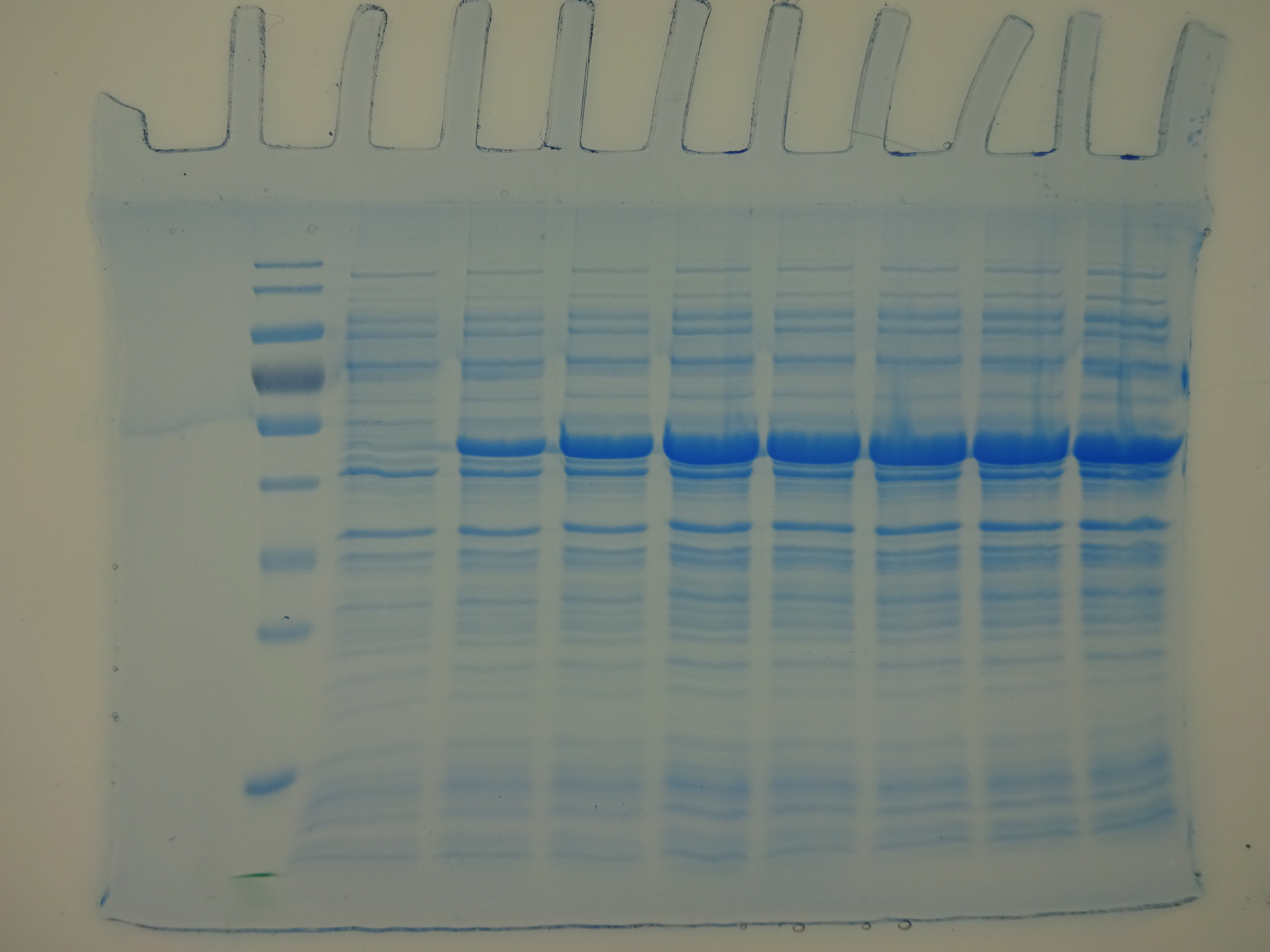

Supplement: Supplementary file 1 [file Data_Sheet_1.zip › new figs/Raw Image of Figure 2-Panel A.tif]

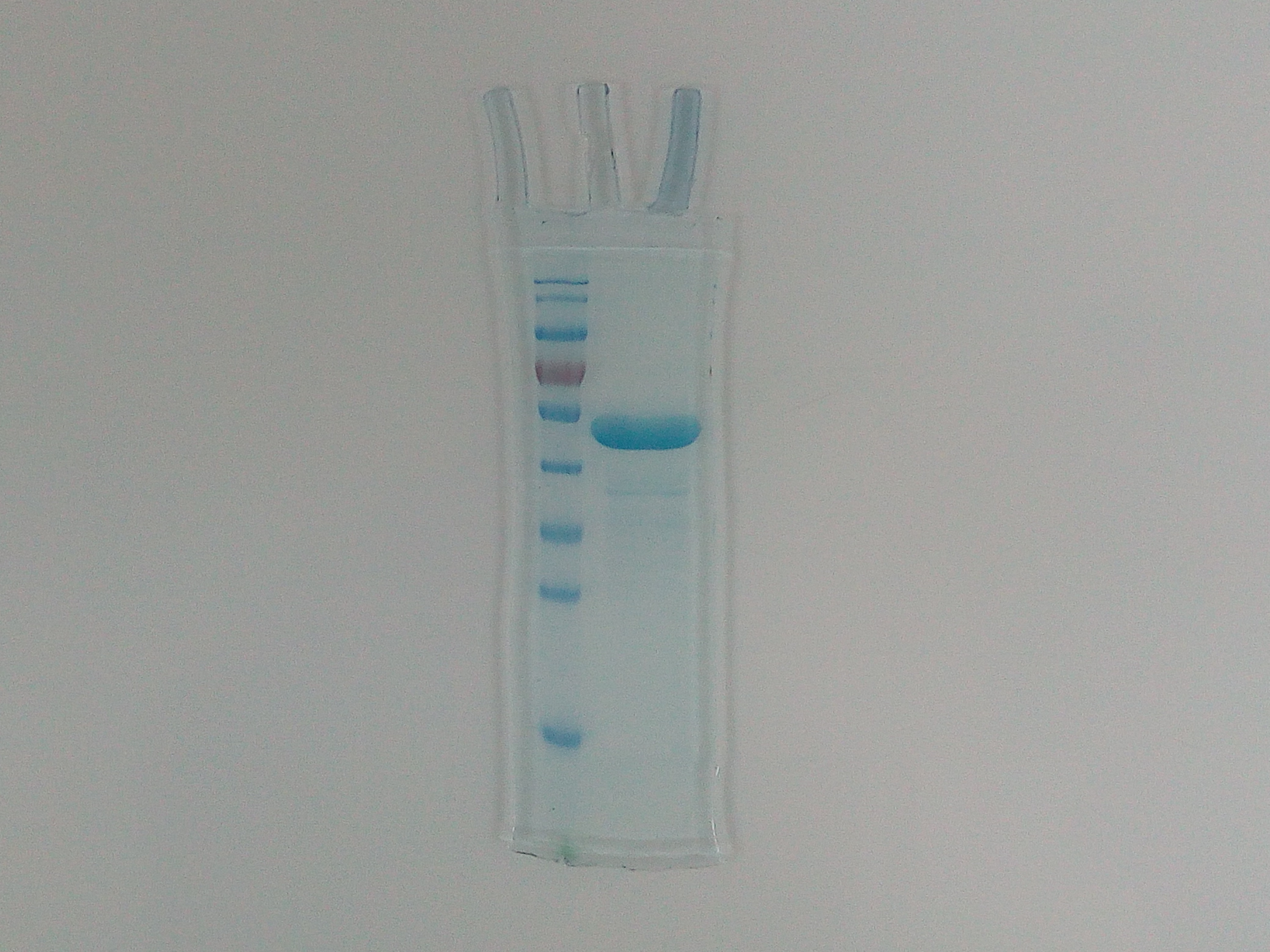

Supplement: Supplementary file 1 [file Data_Sheet_1.zip › new figs/Raw Image of Figure 2-Panel B.jpg]

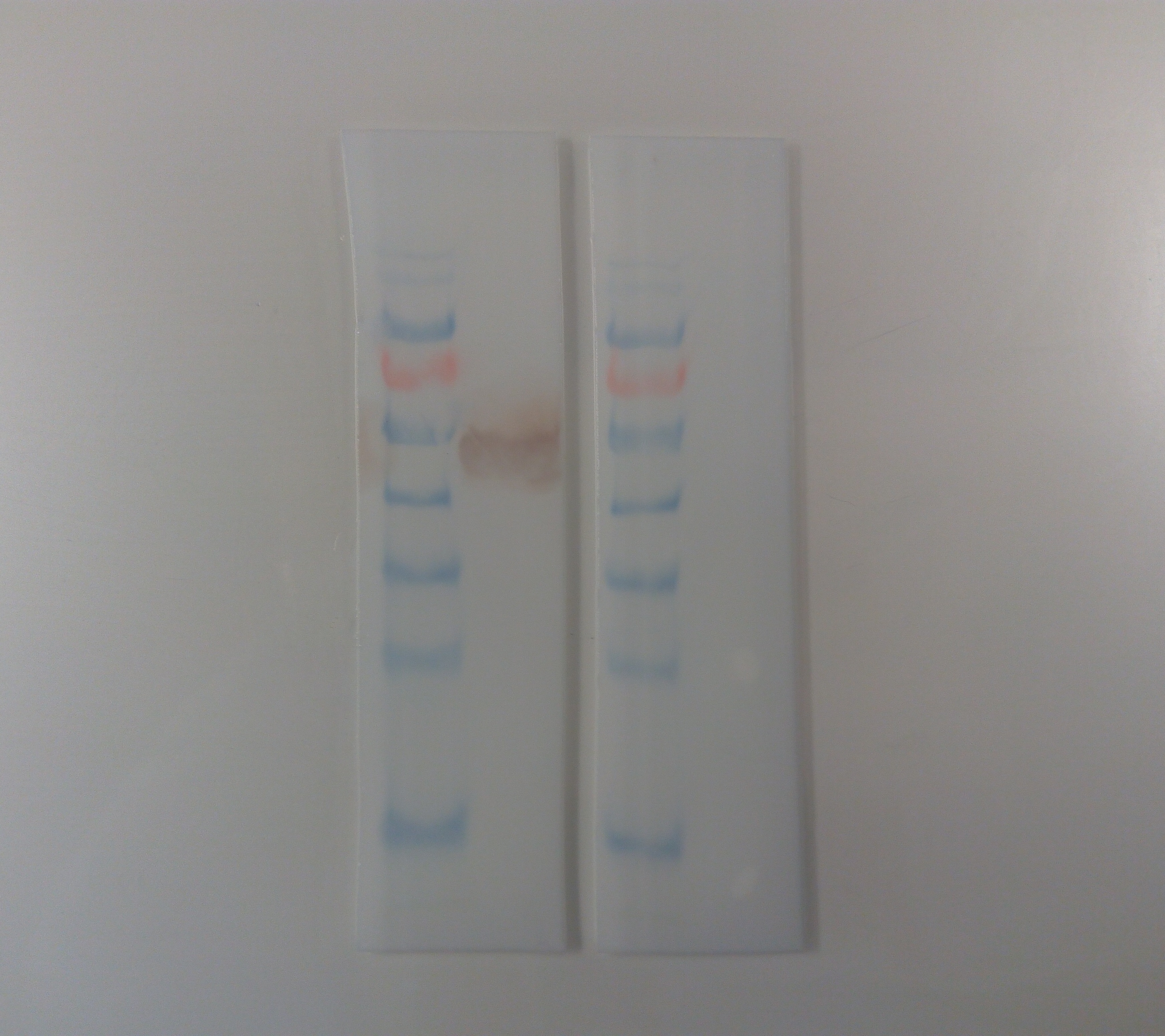

Supplement: Supplementary file 1 [file Data_Sheet_1.zip › new figs/Raw Image of Figure 2-Panel C.jpg]

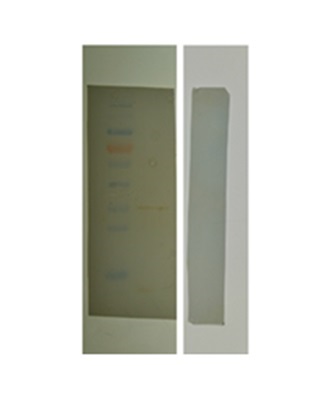

Supplement: Supplementary file 1 [file Data_Sheet_1.zip › new figs/Raw Image of Figure 2-Panel D.jpg]
