# Supplementary material for: The Serine/Threonine-Protein Phosphatase 1 From Haemonchus contortus Is Actively Involved in Suppressive Regulatory Roles on Immune Functions of Goat Peripheral Blood Mononuclear Cells
Source: Front Immunol. 2018 Jul 16;9:1627. doi: 10.3389/fimmu.2018.01627 (PMC6054924; doi:10.3389/fimmu.2018.01627)
Supplement: Supplementary file 2 [file Image_1.PDF]

Figure S1:

Phylogenetic analysis by neighbour joining method using MEGA 6.1 software. The cladogram was constructed for amino acid sequences of HcSTP-1 to that of other known STPs, which showed that all sequences had much similar structural makeup.

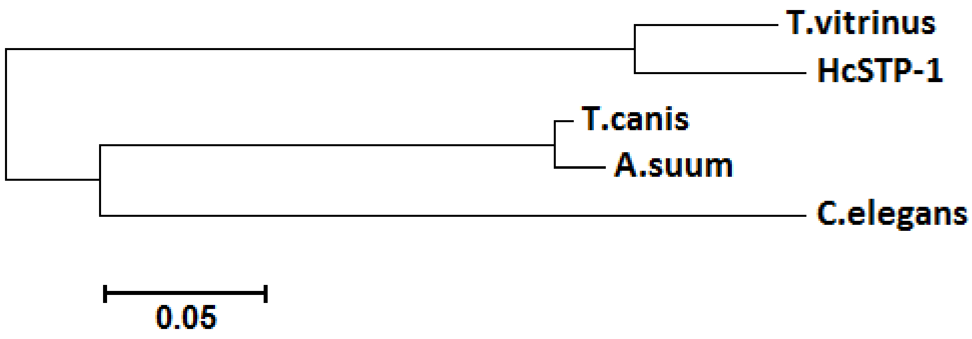

Figure S2:

The protein sequence of *H. contortus* STP-1 protein was used to predict N-terminal signal peptides by SignalP 4.1 Server (http://www.cbs.dtu.dk/services/SignalP/), which showed lake of any SP in its structure.

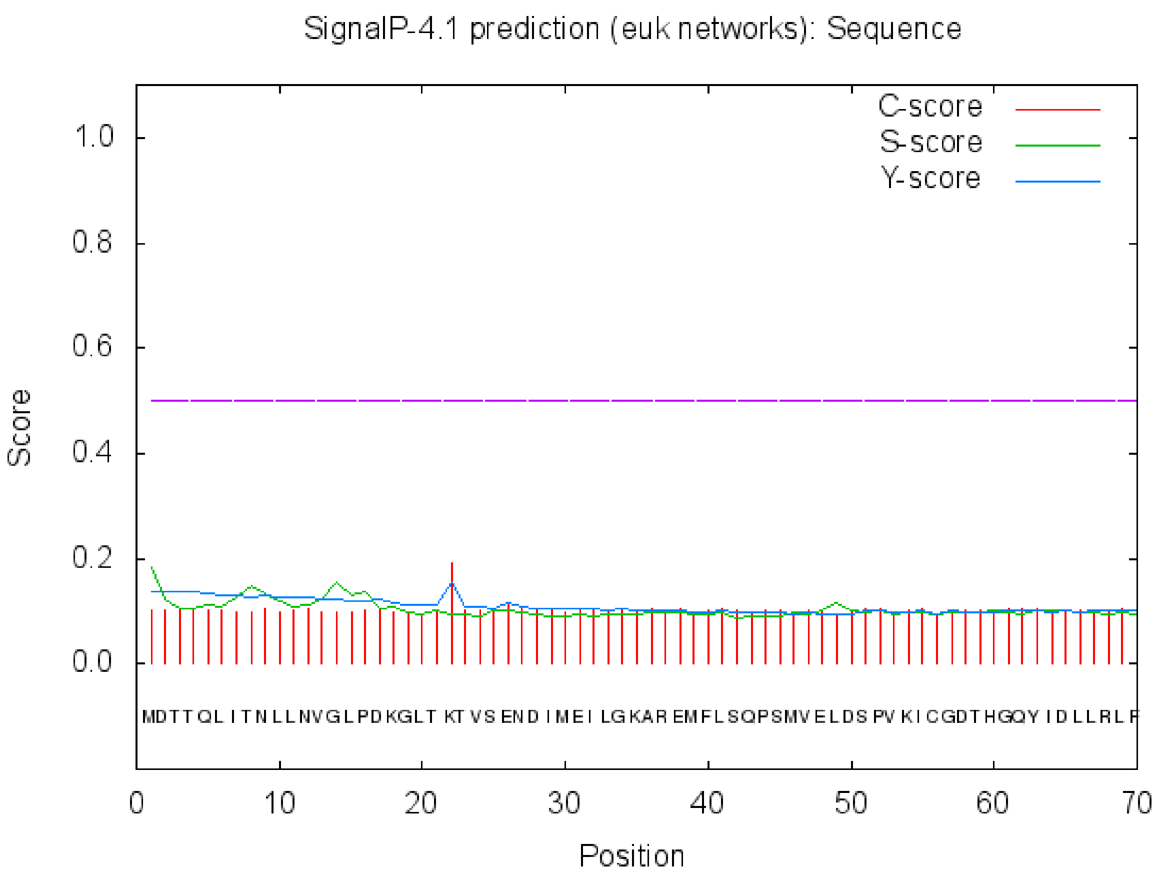

Figure S3:

Membrane protein prediction using TMHMM Server v.2.0. The amino acid sequences of HcSTP-1 was analysed to predict transmembrane structures using TMHMM Server v.2.0. There was no transmembrane domains was predicted in this protein structure. (http://www.cbs.dtu.dk/services/TMHMM/).

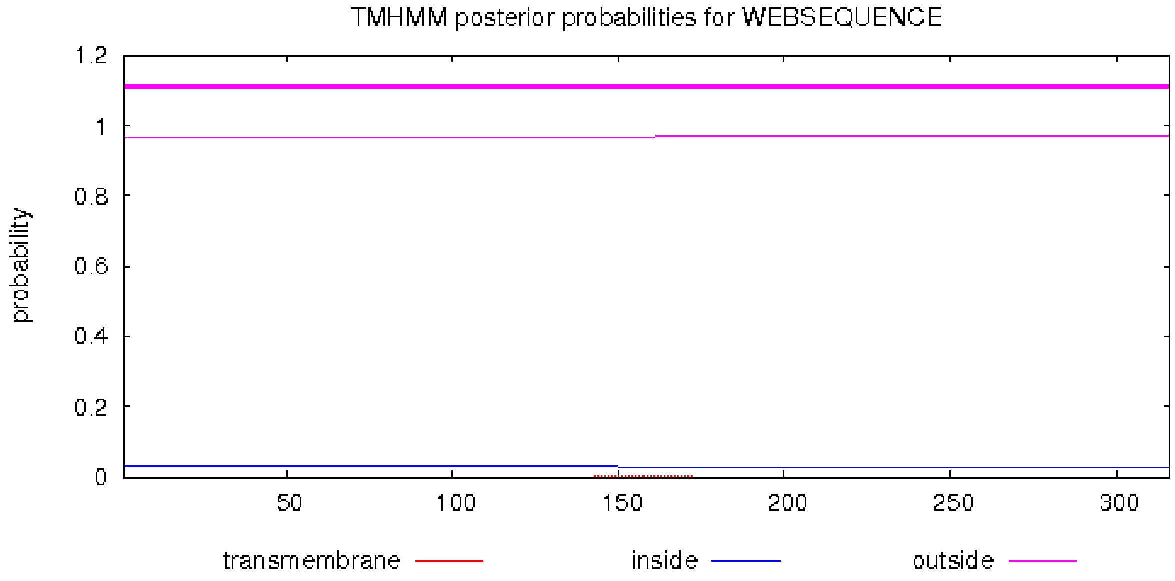

Figure S4:

Prediction of T and B cell epitopes: Protein sequence of HcSTP-1 was used for the prediction of the T cell and B cell epitopes, that revealed 13 peptides of T cell epitopes and 18 B cell epitopes in protein structure.

T. cell Motifs:

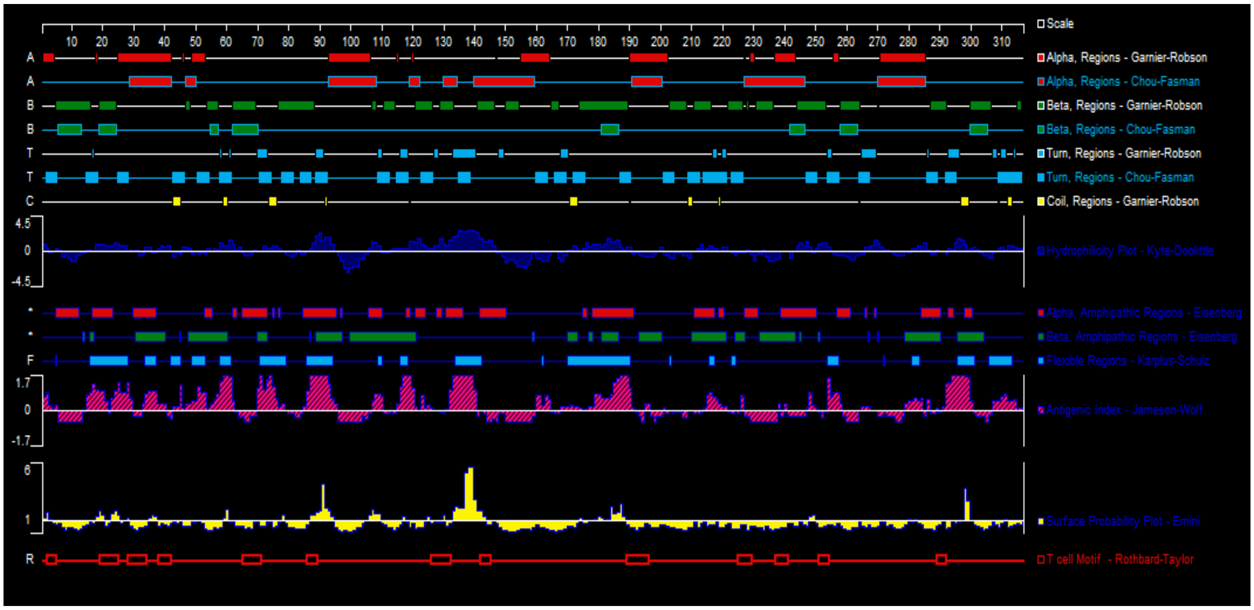

No.	Start	End	Epitopes	Length
1	2	5	DTTQ	4
2	19	25	GLTKTVS	7
3	28	34	DIMEILG	7
4	38	42	EMFLS	5
5	65	71	DLLRLFN	7
6	86	89	DYVD	4
7	126	132	RAYGFYE	7
8	142	145	RMWQ	4
9	189	196	DALGATLE	8
10	225	229	DILAK	5
11	237	241	DLVAR	5
12	251	254	EFFG	4
13	289	292	DVFR	4

B. Cell Epitopes:

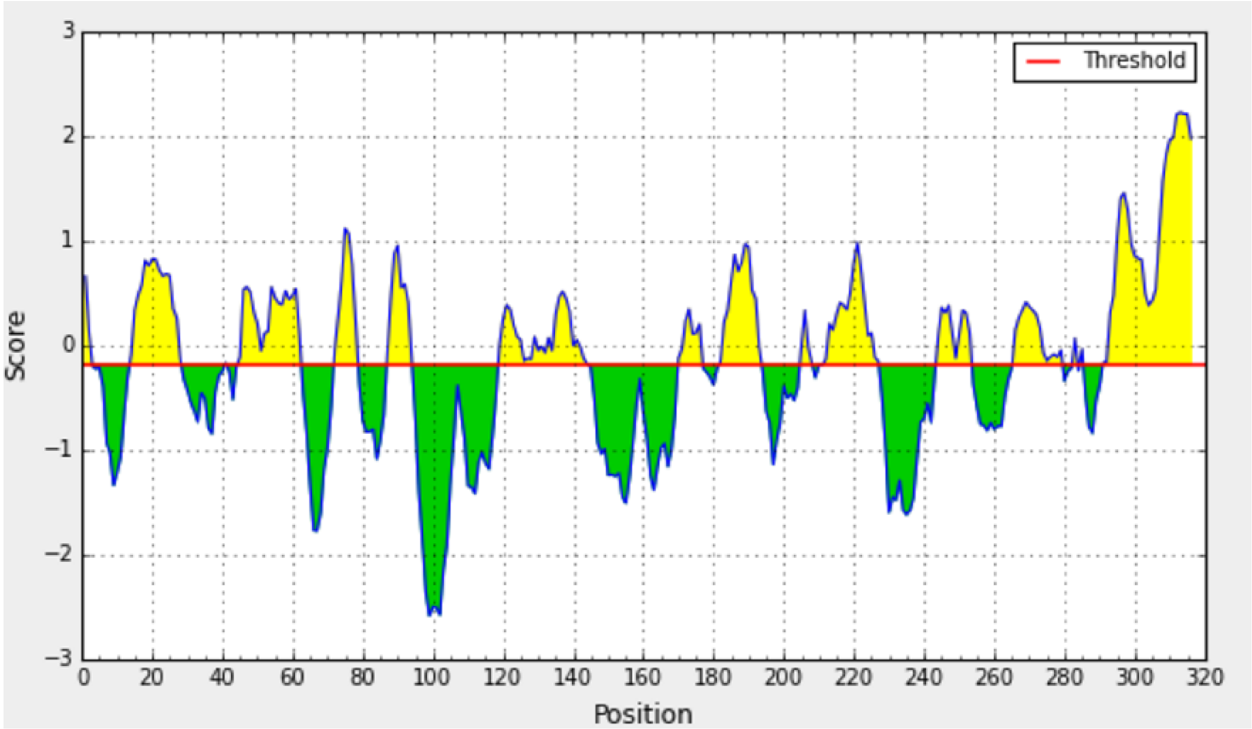

No.	Start	End	Epitopes	Length
1	1	2	MD	2
2	15	27	LPDKGLTKTVSEN	13
3	46	50	MVELD	5
4	52	62	PVKICGDTHGQ	11
5	73	78	GGFPPL	6
6	88	93	VDRGKQ	6
7	119	125	HECANVN	7
8	129	129	G	1
9	133	133	E	1
10	135	141	NRRYQSQ	7
11	172	176	SPHLQ	5
12	183	192	NITRPTDALG	10
13	206	206	I	1
14	213	225	ANIRGASYGFGPD	13
15	244	248	QVVQD	5
16	250	253	YEFF	4
17	266	273	HYCGQFDN	8
18	283	283	N	1
