# Supplementary material for: The Serine/Threonine-Protein Phosphatase 1 From Haemonchus contortus Is Actively Involved in Suppressive Regulatory Roles on Immune Functions of Goat Peripheral Blood Mononuclear Cells
Source: Front Immunol. 2018 Jul 16;9:1627. doi: 10.3389/fimmu.2018.01627 (PMC6054924; doi:10.3389/fimmu.2018.01627)
Supplement: Supplementary file 3 [file Table_1.PDF]

The real-time PCR reactions and conditions:

The real-time PCR reactions were carried out in 96-well optical reaction plates (BioRad Laboratories, CA, USA) with 200 nM each specific primer, 2 μl cDNA (20 ng, as described in the experimental procedures), and SYBR® Green Supermix (BioRad Laboratories, CA, USA) using an ABI 7500 Real-Time PCR system (Applied Biosystems, CA, USA). The PCR conditions were as follows: 95°C for 30 sec, followed by 95°C for 15 sec, 60°C for 30 sec and 72°C for 30 sec for 40 cycles.

Supporting Tables:

Table S1. Primers sequences for real-time PCR.

Gene Name	Primer Sequence (5’-3’)	Gene length (bp)	Amplification efficiency (%)*	Correlation coefficients (r^2)
β-actin	CACCACACCTTCTACAAC<br>TCTGGGTCATCTTCTCAC	106	95.41	0.9991
IL-2	CAAACGGTGCACCTACTTCA<br>AGCTTGAGGTTCTCGGGATT	115	96.75	0.9985
IL-6	CGTCGACAAAATCTCTGCAA<br>TTCCCTCAAACTCGTTCTGG	148	98.73	0.9994
IL-10	CCTTGTCGGAAATGATCCAG<br>AGGGCAGAAAACGATGACAG	150	98.68	0.9993
TGF-β1	GAACTGCTGTGTTCGTCAGC<br>TCCAGGCTCCAGATGTAAGG	126	98.98	0.9996
IFN-γ	GAACGGCAGCTCTGAGAAAC<br>GGTTAGATTTTGGCGACAGG	131	98.02	0.9982
IL-17	TTGTAAAGGCAGGGGTCATC<br>GGTGGAGCGCTTGTGATAAT	149	96.68	0.9978

* Amplification efficiency (%) = $(10^{-1/slope} -1) \times 100$

Table S2. Reagents for real-time PCR

Reagent	Volume
2×ChamQSYBR qPCR Master Mix	10.0 μl
Primer 1 (10μM)	0.4 μl
Primer 2 (10μM)	0.4 μl
50×ROX Reference Dye 2 (50×)	0.4 μl
cDNA	X μl
ddH_2O	Total= 20.0 μl

Table S3. Cyclic conditions for real-time PCR.

Stage 1	Pre-degeneration	Reps: 1	95 °C	30 sec
Stage 2	Circular reaction	Reps: 40	95 °C	10 sec
			60 °C	30 sec
Stage 3	Melt curve	Reps: 1	95 °C	15 sec
			60 °C	60 sec
			95 °C	15 sec
